# Clinical Applications of Iso-Inertial, Eccentric-Overload (YoYo™) Resistance Exercise

**DOI:** 10.3389/fphys.2017.00241

**Published:** 2017-04-27

**Authors:** Per A. Tesch, Rodrigo Fernandez-Gonzalo, Tommy R. Lundberg

**Affiliations:** ^1^Department of Physiology & Pharmacology, Karolinska InstitutetStockholm, Sweden; ^2^Radiobiology Unit, Laboratory of Molecular and Cellular Biology, Belgian Nuclear Research Centre, Institute for Environment, Health and Safety, SCK•CENMol, Belgium; ^3^Department of Laboratory Medicine, Division of Clinical Physiology, Karolinska Institutet, and Unit of Clinical Physiology, Karolinska University HospitalStockholm, Sweden

**Keywords:** clinical trials, eccentric training, flywheel exercise, rehabilitation, skeletal muscle, strength training

## Abstract

In the quest for a viable non-gravity dependent method to “lift weights” in space, our laboratory introduced iso-inertial resistance (YoYo™) exercise using spinning flywheel(s), more than 25 years ago. After being thoroughly tested in individuals subjected to various established spaceflight analogs, a multi-mode YoYo™ exercise apparatus was eventually installed on the International Space Station in 2009. The method, applicable to any muscle group, provides accommodated resistance and optimal muscle loading through the full range of motion of concentric actions, and brief episodes of eccentric overload. This exercise intervention has found terrestrial applications and shown success in enhancing sports performance and preventing injury and aiding neurological or orthopedic rehabilitation. Research has proven that this technique offers unique physiological responses not possible with other exercise hardware solutions. This paper provides a brief overview of research that has made use, and explored the efficacy, of this method in healthy sedentary or physically active individuals and populations suffering from muscle wasting, disease or injury. While the collective evidence to date suggests YoYo™ offers a potent stimulus to optimize the benefits of resistance exercise, systematic research to support clinical use of this method has only begun to emerge. Thus, we also offer perspectives on unresolved issues, unexplored applications for clinical conditions, and how this particular exercise paradigm could be implemented in future clinical research and eventually being prescribed. Fields of particular interest are those aimed at promoting muscle health by preventing injury or combating muscle wasting and neurological or metabolic dysfunction due to aging or illness, or those serving in rehabilitation following trauma and/or surgery.

## Introduction

The National Aeronautics Space and Administration (NASA) and the international space medicine community recognized muscle loss and associated neuromuscular dysfunction following spaceflight as serious concerns decades ago, challenging the prospect of future travels to distant planets. Although it was long known that low intensity aerobic exercise does not produce muscle hypertrophy or increased muscle strength or power, for many years the idea prevailed that any aspect of physical deconditioning, typical of spaceflight, could be attenuated by prescribing in-flight aerobic exercise (Convertino, 1994). In the non-peer-reviewed technical journal Griffin, published in 1990 by the SAAB-SCANIA group, Drs. Dudley and Tesch discussed different tentative solutions, including “blue-sky” concepts that could battle space-induced muscle atrophy and dysfunction. Not leaving out pharmacological or nutritional interventions, these authors concluded that resistance exercise presents by far the most attractive solution to cope with and offset neuromuscular dysfunction that occurs with long-term exposure to microgravity. However, lack of (or reduced) gravity forces on a space vehicle, station or distant planet, limits or excludes the use of the vast majority of effective commercial exercise apparatuses. As a consequence, a finite solution regarding exercise hardware and prescriptions to combat such effects *in-flight* is still not routinely operational.

In addition to being responsible for producing body movement(s), skeletal muscle, by far the largest organ of the human body, plays an essential role as a reservoir for, and regulator of, metabolic and endocrine processes. Hence, preserving muscle and neuromuscular function is critical to health and quality of life (Doherty, 2003). Consequently, muscle loss resulting from spaceflight, but also from aging, disuse, trauma or disease, presents a serious threat to skeletal muscle integrity and health. As resistance exercise serves as the cardinal, and unchallenged, non-pharmaceutical strategy to combat deleterious effects on skeletal muscle function and mass, pursuing highly effective exercise hardware and protocols are warranted. Following the introduction of the iso-inertial YoYo™ technology as a viable and physiologically sound exercise device for space crew (Berg and Tesch, 1994), mounting evidence suggests this solution offers, and mediates, unique physiological responses and benefits beyond what is possible by means of other established resistance exercise interventions (Norrbrand et al., 2008, 2010, 2011; Cuenca-Fernandez et al., 2015). There is sparse, yet growing evidence that this method also has important clinical applications and could serve geriatric populations and athletes and other patients in need of rehabilitation following muscle wasting or orthopedic/neurological trauma (Greenwood et al., 2007; Onambele et al., 2008; Romero-Rodriguez et al., 2011; Fernandez-Gonzalo et al., 2014c; Sarmiento et al., 2014; Abat et al., 2015; Oliveira et al., 2015; Gual et al., 2016; Fernandez-Gonzalo et al., 2016a). This review covers research, pertinent to clinical applications, which has made use of, and explored the efficacy of, this novel resistance exercise paradigm favoring eccentric overload. Other exercise methods and technologies which offer and emphasize eccentric loading profiles different from “normal” coupled concentric and eccentric action cycles, as in lifting and lowering a weight, or electrically powered devices, have been employed in research and clinical settings. It is beyond the purpose of this paper to review the literature covering the topic of eccentric exercise training. For this, the reader is referred to other excellent reviews (e.g., LaStayo et al., 2003, 2014; Hedayatpour and Falla, 2015) providing very strong support for eccentric exercise training, including accentuated eccentric loading, as a highly effective, safe and feasible clinical method.

## The evolution of iso-inertial YoYo™ exercise

The birth of the YoYo™ evolved from several pivotal research findings and challenges. First and foremost, there was an urgent quest for a non-gravity dependent method to mimic weight lifting and serving astronauts in space while coping with associated operational, physiological and technical challenges. The confined environment on any spacecraft calls for an exercise apparatus that possesses a small envelope and mass that could readily be dismounted and stowed. Materials selection of the components of any product flown on a spaceship is restricted, as e.g., numerous materials are considered hazardous. Ideally, the exercise modality chosen should not use excess energy, water, O_2_, or external power, as supply of these sources is restricted on a spacecraft.

Past research using a motor-driven device had shown that elbow flexor resistance exercise with eccentric actions produced greater increases in muscle strength than exercise using concentric actions (Komi and Buskirk, 1972). Spurred by the results of this particular study and other subsequent observations using exercise(s) emphasizing eccentric actions (Häkkinen and Tesch, 1981), we initiated a series of studies in the late 1980's employing either motor-driven (Tesch et al., 1990) or weight-loaded resistance exercise (Hather et al., 1991; Dudley et al., 1991a,b) to explore the effects of combinations of coupled eccentric and concentric training programs on muscle size, morphology and metabolism as well as motor function. Collectively, in healthy men and women, constant velocity (i.e., isokinetic) resistance exercise involving eccentric actions produced more robust increases in muscle strength than concentric resistance exercise (Colliander and Tesch, 1990, 1991). However, these changes were accompanied by modest muscle hypertrophy. In contrast, chronic coupled concentric-eccentric resistance exercise training with heavy weights, calling for acceleration and deceleration, prompted significantly greater muscle hypertrophy and increases in strength, than training omitting eccentric actions (Hather et al., 1991; Dudley et al., 1991b). Likewise, and regardless of exercise modality, eccentric training carried over to favorable adaptations in concentric modes, whereas concentric actions had less impact on eccentric strength and performance outcomes (Colliander and Tesch, 1990, 1991; Dudley et al., 1991b). It is also noteworthy that rate of improvements and progression in training load over the course of training tended to plateau much earlier when eccentric actions were left out.

To our knowledge, the history of using the inertia of flywheel(s) as a source to provide resistance for muscular work dates back to 1796 when Frenchman Francis Lowndes described a whole-body exercise device based on this principle (Good et al., 1819). While this apparatus was aimed at executing physiotherapeutic exercises, giant scientists and Nobel Prize laureates A.V. Hill and August Krogh and associates independently conducted experiments based on this mechanical principle in the first decades of the twentieth century (Krogh, 1913). However, the pioneer work by these entrepreneurs and scientists, similar to those by investigators in more recent times (Pearson et al., 2001), focused on exploring bioenergetics and mechanics of skeletal muscle, rather than utilizing this tool to offer exercise training.

The paper by Berg and Tesch, published in 1994, re-introduced and described how inertia of a spinning flywheel(s) could be utilized to execute resistance exercise. Again, this work emanated from the quest and challenge of “lifting weights” to serve astronauts on missions in space. Consequently, novel hardware (the YoYo™ Leg Press configuration) was tested and validated by the crew who completed the STS-78 Life Science mission on Space Shuttle Columbia in 1996 (Tesch et al., 2005). A year earlier, and in preparation for this important spaceflight, eight healthy men were examined over several weeks of bed rest and subjected to YoYo™ exercise testing (NASA, 1996). Subsequent experiments, sponsored by NASA and the European, Canadian, Russian and Japanese space agencies, explored the effects of YoYo™ training on muscle and bone of men and women subjected to various analogs simulating spaceflight (e.g., confinement, bed rest or unilateral lower limb suspension up to 110 days; see below).

## The YoYo™ method

**Figure 1:**
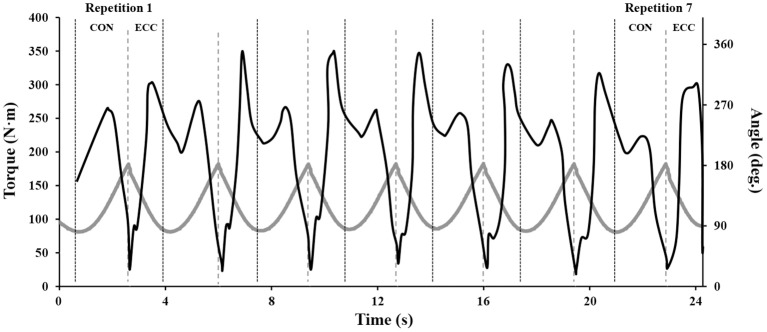
**Typical smoothed torque values across a set of 7 maximal repetitions of YoYo™ knee extension exercise in young healthy men**. Black line denotes torque; gray line denotes knee angle. Typically, torque increases over the initial three repetitions, whereas from the fourth or fifth repetition, peak torque typically decreases due to fatigue.

In brief, any apparatus or configuration using the current principle (Berg and Tesch, 1994; Norrbrand et al., 2008) is the resemblance of a reversed children's toy yoyo (hence the trademark YoYo™). Pulling, pushing or curling the strap, which is anchored to a pivoting lever arm of the apparatus, a handle attachment, or a harness worn by the trainee (Alkner et al., 2003; Norrbrand et al., 2011), initiates rotation of the fixed shaft, which holds the flywheel(s). The inertia is determined by mass, configuration/profile and diameter of the wheel(s). Once the strap is fully wound off the shaft, the energy imparted and stored in the system pulls the strap back onto the shaft, as the trainee aims at resisting this action, bringing the flywheel(s) to a stop and thus completing a cycle. Unlike in lifting a weight, this unique exercise method ensures accommodated resistance, and provided effort is maximal, optimal muscle loading at any particular joint angle through the entire concentric action. This way, and because there is no “sticking point” (or biomechanical lever issues) involved when performing YoYo™ exercise, energy or work produced exceeds by far what can be attained in the concentric action using gravity dependent free weights. The subsequent eccentric action must then absorb the energy stored in the flywheel(s). Due to the absence of, or minute, frictional force characterizing any YoYo™ system, the energy for the concentric and eccentric phases will be almost identical. However, by delaying and hence performing the breaking action in a very narrow window, the time to dissipate the kinetic energy produced is condensed. This approach results in greater eccentric peak force and power (energy/time). Thus, eccentric “overload” is offered by strategically and intentionally delaying initiation of the breaking action (Figure 1). The protocols employed in the vast majority of studies, referred to in this review, used four sets of seven repetitions using maximal effort and the above strategy, and had volunteers perform this regime either every third day or, alternately, 2 or 3 days weekly.

While the execution of resistance exercise using free weights (e.g., barbell, dumb bell, kettle bell) certainly offers several attractive and versatile features, YoYo™ possesses additional unique attributes facilitating physiological advantages over free weights. Assuming the effort is maximal in the very first repetition of a set and thereafter, all repetitions produce maximal loading through the full range of motion of the concentric action (see above). When lifting a weight for a set to failure, or when using other accentuated eccentric training forms where the load is set, only the last repetition calls for maximal activation, yet only at the angle representing the sticking point. Also, without assisted spotting, free weight exercise cannot readily provide eccentric overload. Similar to when lifting a free weight, but in contrast to what is the case with most commercial weight loaded exercise systems (i.e., weight stacks), there is negligible frictional forces involved in propelling the flywheel(s) of the YoYo™ to accelerate or decelerate the motion. Indeed, the vast majority of commercial weight stack machines inherently produce marked eccentric “under-load” due to excess friction (Dudley et al., 1991b).

## Acute responses to YoYo™ exercise

Similar to other resistance exercise modalities (Dudley et al., 1991a), YoYo™ is performed at a very low metabolic cost (Berg and Tesch, 1994; Caruso and Hernandez, 2002). This is because the long known fact that energy needed to perform an eccentric action is only about one fifth of that required for the concentric action of the same cycle (Fenn, 1924). Hence, populations suffering from, or at risk of, cardiovascular pathologies could benefit from any exercise favoring eccentric actions, and this is certainly advantageous on missions in orbit where energy is to be preserved.

In contrast to running, jumping and hopping activities, characterized by a short-lived eccentric action initiating the stretch-shortening cycle (SSC), any YoYo™ exercise relies on both prolonged eccentric exposure and a brief episode of very high SSC activity (Martinez-Aranda and Fernandez-Gonzalo, 2016). In the world of sports, alpine skiing is unique as the eccentric action critical in making the skis to turn, is executed at a very slow angular velocity (Berg et al., 1995; Tesch, 1995). As an example, and similar to exercise using YoYo™, the giant slalom calls for greater eccentric than concentric EMG activity.

Studies comparing surface EMG during all-out exercise using weights or weight-stack machines on one hand and YoYo™ in a given exercise task consistently report greater global EMG activity with YoYo™ (Berg and Tesch, 1994; Onambele et al., 2008; Norrbrand et al., 2010). Likewise, in head-to-head comparisons assessing muscle involvement by means of functional magnetic resonance imaging (fMRI), the YoYo™ Supine Squat (Norrbrand et al., 2011) and YoYo™ Leg Press (Berg et al., 1991) produced significantly greater muscle use than the barbell squat. These notions were confirmed in professional soccer players, who were subjected to acute exercise using four different hamstring modalities (Fernandez-Gonzalo et al., 2016b; Mendez-Villanueva et al., 2016). By employing fMRI analysis, these investigators showed greater involvement of all aspects of the hamstring muscle complex, including agonists, and more substantial individual muscle use with the YoYo™ Leg Curl compared with other established gravity or non-gravity dependent hamstring exercise methods examined. The superior efficacy to involve the hamstring muscles substantiates the findings of Tous-Fajardo et al. (2006), who measured EMG activity in twenty rugby and soccer players performing bouts on the YoYo™ Leg Curl. Thus, surface EMG activity of mm. biceps femoris and semitendinosus, most prone to hamstring injury, was significantly higher than EMG measured during maximal voluntary contractions (MVCs) of the knee flexors. Given that any exercise-induced enhancement in muscle signal intensity (T_2_), assessed by means of fMRI, is proportional to EMG activity (Adams et al., 1992) and work (Jenner et al., 1994), it appears YoYo™ exercise facilitates more substantial muscle involvement than other resistance exercise methods.

Another intriguing effect elicited by the method was reported by Cuenca-Fernandez et al. (2015). Competitive swimmers carried out one bout of 3–4 lunges using either the YoYo™ Squat or barbell 8 min prior to a sprint swim, to determine and compare effects on the post-activation potentiation (PAP) phenomenon (Hodgson et al., 2005). This acute effect, reflected in augmented contractile force, is believed to be due to amplified myosin cross-bridge activation mediated by increased myoplasmic Ca^2+^ concentrations. Interestingly, the YoYo™ intervention enhanced neuro-motor performance more than the barbell task. Thus, this acute sole bout had a most significant impact as speed and hence swim sprint performance was improved. The authors attributed this response to the PAP effect.

Altogether, the greater overall load and mechanical stress placed on muscle, eliciting more concentric and as a result more eccentric work, appears to be responsible for the more robust muscle use demonstrated with YoYo™ exercise. Additionally, we speculate other unidentified supraspinal and spinal mechanisms facilitate motor unit recruitment beyond what is conceivable by means of standard weight-loaded exercise or other accentuated eccentric loading forms.

## Exercise training benefits in healthy individuals and athletes

The premier training study that employed the open-chain knee extensor YoYo™ (12 sessions over 5 weeks) reported increased isometric, concentric and eccentric force or strength accompanied by a 6% increase in quadriceps muscle volume (Tesch et al., 2004a). Taken together with the results of subsequent studies employing similar protocols (Table 1), weekly increases in force production typically exceed 2%. This effect is paralleled by increases in muscle size of ~1% per week in response to short-term (~5 weeks) training programs (Seynnes et al., 2007; Norrbrand et al., 2008; Lundberg et al., 2013). Whereas traditional knee extensor weight-stack training shows comparable strength increases (Norrbrand et al., 2008), muscle hypertrophy occurs at a rate that is at least two-fold greater with YoYo™ than with traditional resistance exercise protocols and hardware (Wernbom et al., 2007; Norrbrand et al., 2008). Moreover, 10–20 day YoYo™ training produced significantly increased muscle fascicle length and pennation angle paralleling muscle hypertrophy (Seynnes et al., 2007). Hence, muscle architectural changes may contribute to the early onset of increased force producing capacity. Similar to weight resistance exercise, strength and power increases following YoYo™ chronic exercise training are accompanied by marked neural adaptations evident by increased maximal EMG activity (Seynnes et al., 2007; Norrbrand et al., 2010). This effect is most obvious for eccentric actions and greater with YoYo™ than experienced with exercise using weights (Norrbrand et al., 2008, 2010). It remains to be shown if such an obvious difference in response results from the unique loading features characterizing YoYo™ exercise.

**Table 1:** **Summary of chronic resistance training studies employing YoYo™**.

**Study**	**Design**	**Subjects**	**Exercise protocol**	**Key results**	**Comment**
**HEALTHY INDIVIDUALS**					
Tesch et al., 2004a	5 week; single group.	10 ♂ and ♀ (30–53 year).	4 × 7 YoYo™ knee extensions; 2–3 × week^−1^.	MVIC ↑~12% QF CSA ↑~6% EMG activity ↔	YoYo™ exercise showed increased strength and marked hypertrophy.
Seynnes et al., 2007	35 day; parallel groups; controls did not train.	7 ♂ and ♀ (20 ± 2 year); 6 controls (22 ± 3 year).	4 × 7 YoYo™ knee extensions; 3 × week^−1^.	MVIC ↑ 39% EMG activity↑ 35% QF CSA ↑~7% Fascicle length ↑ 10% Pennation angle ↑ 8%	YoYo™ induced greater early structural adaptations than reported in the bulk of literature.
Norrbrand et al., 2008, 2010	5 week; parallel groups (YoYo™ vs. WS exercise).	YoYo™ 7 ♂ (39 ± 9 year). WS: 8 men 39 ± 8 year).	4 × 7 YoYo™ knee extensions or WS, 2–3 × week^−1^.	CSA: YoYo™ ↑ 6%, ↑ WS 3% MVIC: YoYo™ ↑ 8%, WS ↑ 5%	More robust adaptations with YoYo™ compared with conventional WS.
Lundberg et al., 2013	5 week; unilateral design, one leg did YoYo™ only.	10 recreationally active ♂ (25 ± 4 year).	4 × 7 YoYo™ knee extensions, 2–3 × week^−1^.	CSA ↑ 8% MVIC ↑ 11% Training-specific strength ↑ 29%	YoYo™ exercise produced increased strength and marked hypertrophy.
Lundberg et al., 2014	5 week; unilateral design, one leg did YoYo™ only.	10 moderately trained ♂ (26 ± 5 year).	4 × 7 YoYo™ knee extensions, 2-3 × week^−1^.	CSA ↑ 3% Training-specific strength ↑ 12% MVIC ↔	YoYo™ exercise produced increased strength and muscle hypertrophy.
Fernandez-Gonzalo et al., 2014c	6 week; parallel groups (♂ vs. ♀).	32 ♂ and ♀ (24 ± 1 year).	4 × 7 YoYo™ supine squats, 2–3 × week^−1^.	1-RM ↑ 25% in ♂, ↑ 20% in ♀ Thigh CSA ↑ 5% both groups Jump height ↑both groups (4–8%) Peak power ↑ 10–20% both groups Markers of muscle damage elevated after the first, but not the last bout	YoYo™ supine squat elicited comparable and favorable gains in strength, power and muscle mass in both ♂ and ♀. Muscle damage did not hinder these adaptations.
Tous-Fajardo et al., 2016	11 week; parallel groups (YoYo™ and vibration vs. conventional).	24 ♂ soccer players (17 ± 0.5 year).	YoYo™ 2 × 6–10 squats + unilateral vibration squats, 1 × week^−1^. Conventional: Weight-training + linear speed training, 1 × week^−1^.	Both groups ↑ sprint and jump performance COD ↑in the YoYo™ group only	YoYo™ squat and vibration exercise could improve soccer-specific performance.
**SPACE-RELATED**					
Alkner et al., 2003	YoYo™ RE performed during 110 day of simulated space station confinement.	4 ♂ (27–45 year).	YoYo™ 4 × 10 calf raise, squat, back extension, seated row, lateral shoulder raise, biceps curl, 2–3 × week^−1^.	Training load ↑ (range 16–108%) during the confinement period. MVIC ↔	YoYo™ maintained or increased performance during long-term confinement.
Alkner and Tesch, 2004a	29 day; bed rest with (*n* = 8) or without (*n* = 9) YoYo™ RE.	17 ♂ (range 26–41 year).	YoYo™ 4 × 7 supine squats and 4 × 14 calf press 2–3 × week^−1^.	With bedrest: ↓ QF (−10%) and TS (−16%) CSA With YoYo™ 0% (QF) and −8% (TS)	YoYo™counteracted muscle atrophy inflicted by bed rest.
Alkner and Tesch, 2004b; Alkner et al., 2016; Rittweger et al., 2005; Nielsen et al., 2016; Irimia et al., 2017	90 day; bed rest with (*n* = 8) or without (*n* = 9) YoYo™ RE.	17 ♂ (range 26–41 year).	4 × 7 YoYo™ supine squats and 4 × 14 calf press 2–3 × week^−1^.	With bedrest: ↓ QF (−18%) and TS (−29%) CSA ↓ Strength and power (−31–60%) ↓ EMG activity (−28–35%). With YoYo™ QF volume ↔ and TS ↓15% MVIC ↓ less than in the bed rest group Bone loss was partly rescued Collagen content ↔ Metabolic perturbations were partly rescued	YoYo™ counteracted muscle atrophy and functional decrements induced by 90 day bed rest in knee extensors. YoYo™ was partly effective for ankle plantar flexors, for bone loss, and for counteracting metabolic perturbations.
Cotter et al., 2015	10 day; unilateral limb unloading (ULLS) with (*n* = 10) or without (*n* = 9) concurrent YoYo™ exercise (ULLS+RE) using the device constructed for both aerobic and RE.	19 ♂ and ♀ (21 ± 3 year).	4 × 7 YoYo™ supine squats and 6 × 16 calf raises 5 times during the 10 day. (Subjects also did aerobic training).	Knee extensor and ankle plantar flexor strength ↑ in the ULLS+RE group but this change was only different than ULLS in the plantar flexors Peak torque ↓ with ULLS but ↑ for knee extensors and attenuated for ankle plantar flexors with ULLS+RE Most molecular markers affected by ULLS, were ↔ with ULLS+RE	The YoYo™ mitigated the negative effects of 10 day ULLS.
Owerkowicz et al., 2016	5 week; single group using the YoYo™ exercise constructed for both aerobic and RE.	17 ♂ and ♀ (22 ± 0.4 year).	4 × 7 YoYo™ supine squats, 2 × week^−1^. (Subjects also did aerobic training).	3-RM ↑ 18% QF CSA ↑ 10% Fiber CSA ↑ 13% Improvements in isokinetic strength (magnitude not expressed)	Robust muscular adaptations following low-volume YoYo™ RE.
Tesch et al., 2004b; Haddad et al., 2005; Fernandez-Gonzalo et al., 2014a	5 week; ULLS with (*n* = 11) or without (*n* = 10) concurrent YoYo™ exercise (ULLS+RE).	21 ♂ and ♀ (range 30–56 year).	4 × 7 YoYo™ knee extensions, 2–3 × week^−1^.	ULLS ↓ 9% in QF CSA. After ULLS+RE, QF CSA ↑ 8%. Strength ↓ 24–32% after ULLS. ULLS+RE showed ↔ strength. ULLS ↓ oxidative markers and ↑ glycolytic expression. ULLS+RE counteracted these changes	YoYo™ offset knee extensor muscle atrophy and produced marked hypertrophy during chronic unloading.
**ELDERLY**					
Bruseghini et al., 2015	8 week; single group design. First 8 week HIT, then 8 week YoYo™ training after 4 month washout.	12 healthy older adults (68 ± 4 year).	Phase 1: HIT-training 7 × 2 min bouts, 3 × week^−1^. Phase 2: 4 × 7 YoYo™ leg press, 3 × week^−1^.	After YoYo™ training: QF CSA ↑ 4% QF volume ↑ 5% Isokinetic strength ↑~10% LDL cholesterol ↓	Cardiovascular benefits from HIT were not abolished by YoYo™, yet complemented by ↑ muscle size and strength.
Onambele et al., 2008	12 week; parallel groups [YoYo™ (*n* = 12) vs. weight lifting (*n* = 12)].	24 older ♂ and ♀ (70 ± 1 year).	Both groups: Progressive increase from 1 × 8 to 4 × 12 reps, 3 × week^−1^.	Peak power ↑ 28% with YoYo™ compared with ↔ with weights. Tendon stiffness ↑ 136% with YoYo™ and ↑ 54% with weights. Postural balance ↑ only with YoYo™	YoYo™ produced better gains in strength, tendon stiffness and balance than regular weight lifting.
**REHAB/INJURY PREVENTION**					
Gual et al., 2016	24 week; parallel groups. Both groups trained but the intervention group received additional YoYo™ training.	81 basket and volleyball players.	4 × 8 YoYo™ squat, 1 × week^−1^.	Squat-CON, Squat- ECC, and CMJ ↑ more with YoYo™ VISA-R, VISA-L did not differ between groups	YoYo™ enhanced lower limb power without triggering patellar tendon complaints.
Romero-Rodriguez et al., 2011	6 week; prospective case-series study. All subjects received the intervention.	10 national level athletes with tendinopathy.	4 × 10 YoYo™ leg press, 2 × week^−1^.	CMJ ↔ ECC force ↑ (90%) VAS ↓ post training VISA ↑ (86%)	YoYo™ improved muscle function and reduced subjective pain in long-lasting patellar tendinopathy.
Abat et al., 2014, 2015	Average of 4.5 week treatment + YoYo™. Prospective study.	33 patients, diagnosed with insertional patellar tendinopathy.	3 × 10 YoYo™ knee extensions, 2 × week^−1^.	VISA-P ↑ 61%	Intra-tissue percutaneous electrolysis combined with YoYo™ offers clinical and functional improvement of patellar tendon.
Askling et al., 2003	10 week; parallel group design. Both groups trained but the intervention group received additional YoYo™ training.	30 soccer players, control (*n* = 15) and training (*n* = 30) group.	4 × 8 YoYo™ knee flexions,1–2 × week^−1^.	Strength: CON ↑ 15% ECC ↑ 19% Sprint time 30 m ↓ 2.4% Prevalence of injury: 13 in total, 3/13 from intervention group and 10/13 from control	YoYo™ prevented injuries and increased sprint performance.
de Hoyo et al., 2015	10 week; parallel groups. Both groups trained but the intervention group received additional YoYo™ training.	36 junior soccer players, control (*n* = 15) and training (*n* = 18).	From 3 × 6 to 6 × 6 YoYo™ knee flexions and half squats, 1–2 × week^−1^.	Severity likely improved (ES 0.59) CMJ very likely improved (ES 0.58) 10 m flying sprint almost certainly improvement (ES 0.95)	YoYo™ reduced muscle injuries and improved soccer tasks such as jumping ability and linear-sprinting speed.
Greenwood et al., 2007	12 week; parallel groups. Control group did regular strength training.	29 patients with unilateral knee injury. Control (*n* = 14), YoYo (*n* = 15).	4 × 10 YoYo™ knee extensions or regular knee extensions, 3 × week^−1^.	Both groups ↑ vastus lateralis CSA, quadriceps strength, neural activation, standing balance and vertical jump performance. No difference between groups	YoYo™ improved knee extensor size and function after knee injury.
Sanz-Lopez et al., 2016	6 week; with parallel groups.	19 subjects. Control (*n* = 10), YoYo™ (*n* = 9).	4 × 7 YoYo™ squats, 2 × week^−1^. Training performed before a 3-day running intervention where both groups ran.	↑ CSA of Achilles tendon and ↑ pennation angle of m. gastrocnemius medialis with YoYo™. Similar changes with running only	YoYo™ squat induced structural adaptations in Achilles tendon and gastrocnemius.
**PATIENTS**					
Fernandez-Gonzalo et al., 2014c	8 week; single group.	12 stroke patients (63 year, 8 year after stroke onset).	4 × 7 YoYo™ leg press, 2 × week^−1^, unilateral (only most-affected leg).	CON & ECC power ↑ in both trained (40%) and untrained (30%) leg ECC torque ↑ in the affected leg (8%) Balance ↑ 7%, gait in short distances ↑ 17% and functional performance ↑17% ↔ spasticity	YoYo™ induced increases in muscle strength and power of the more-affected, trained limb, and also the untrained leg.
Fernandez-Gonzalo et al., 2016a	12 week; parallel groups; controls did not train.	Training group: 14 stroke patients (61 year) Control group: 15 stroke patients (66 year)	4 × 7 YoYo™ leg press, 2 × week^−1^, unilateral (only most-affected leg).	CSA ↑ 9% Force and power ↑ 14–48% Dual-task-performance ↑ 13% ↑ balance (9%) and gait (11%) ↑ executive functions, attention and speed of information processing No changes in controls	YoYo™ was a powerful aid to regain muscle mass and function in individuals with stroke. Concomitant improvements in cognitive functions.
Oliveira et al., 2015	12 week; single group.	24 multiple sclerosis patients (46 yr).	4 × 8 YoYo™ leg press, 2 × week^−1^.	30 s chair stand ↑ 31% Timed-up-and-go ↑ 24%	YoYo™ exercise improved functional capacity of patients with multiple sclerosis.
Sarmiento et al., 2014	12 week; single group.	12 ♀ with Alzheimer's disease (78 year).	3 × 2 min YoYo™ leg press, 2 × week^−1^.	↓of support phase (9%) and ↑ duration of push off phase (3%). ↑ Contraction time and delayed time of ankle muscles.	YoYo™ improved gait performance in patients with Alzheimer's disease.

*CMJ, counter movement jump; CSA, cross-sectional area; CON, concentric; ECC, eccentric; EMG, electromyography; HIT, high-intensity training; MVIC, maximal voluntary isometric contraction; RE, resistance exercise; RM, repetition maximum; VAS, visual analog scale; COD, change of direction; VISA, Victorian Institute of Sports Assessment (VISA) questionnaire*.

The positive benefits of YoYo™ exercise are manifest in athletes as well. For example, 81 volley- and basketball players examined over 24 weeks had just one weekly bout of 4 sets of 8 repetitions of YoYo™ Squat exercise added to the mandatory conditioning regime. These athletes displayed improved strength and vertical jump performance, and more so than players who did not receive this additional treatment (Gual et al., 2016). Likewise, using the YoYo™ Leg Curl configuration targeting the hamstring muscle group, professional footballers showed improved strength, vertical power and horizontal speed after 10 weeks (1–2 times per week) of training compared with their fellow players who maintained normal strength and power training routines (Askling et al., 2003). Similarly, soccer players subjected to YoYo™ Squat and Leg Curl exercise improved vertical and horizontal power (de Hoyo et al., 2015). This investigative team and others (Martinez-Aranda and Fernandez-Gonzalo, 2016) highlighted the importance of individually identifying and employing the optimal inertia that maximizes performance outcomes. Change of direction (COD) ability is a critical feature in soccer, and almost any other team sport played on turf, wooden floor or ice. Using a multi-task exercise paradigm including the YoYo™ Squat for lateral actions, soccer players were examined before and after training to assess the effects on COD speed (Tous-Fajardo et al., 2016). This proof-of-principle study showed the impact on COD ability was paramount in athletes who were subjected to YoYo™ Squat. Athletes who performed a more conventional strength and speed regime showed no such improvement. Although, the YoYo™ Squat offered by far the most powerful exercise task, the investigators were not able to elucidate the critical stimulus responsible for the markedly enhanced performance.

Collectively, the results to date reveal more robust acute physiological responses, and training-induced neuromuscular adaptations following resistance exercise employing iso-inertial YoYo™ compared with traditional weight training. Hence, employing eccentric overload through this method offers a stimulus that is more potent than that offered by gravity-dependent resistance exercise(s). These attributes extend beyond sports performance and might be of even greater significance in populations suffering from neuromuscular dysfunction, metabolic, hormonal or other disorders. Whether the YoYo™ exercise method is superior to other resistance exercise modalities emphasizing eccentric exercise remains to be explored. Nevertheless, cumulated research data and significant anecdotal evidence suggests that the YoYo™ method offers a safe (see below), yet a powerful and highly time-effective strategy to enhance performance, not only in high-caliber athletes but also in sedentary and clinical populations.

The specific mechanism(s) responsible for such seemingly superior adaptations remain to be defined. Nonetheless, the greater overall concentric work and power produced with the method inherently generates more eccentric power, which ought to impact the endpoint training result. Further, it is highly likely YoYo™ exercise relies on greater proprioception and more so than controlled weight lifting (Hedayatpour and Falla, 2015). This may be because each concentric-eccentric cycle calls for high acceleration in the concentric action and deceleration to bring the flywheel(s) to a stop, evoking significant stretch loads and a brief episode of eccentric overload. While these are paramount stimuli to fully activate all motor units, the high force and power production, and the stretch *per se*, are also fundamental in optimizing skeletal muscle protein synthesis (Tannerstedt et al., 2009; Friedmann-Bette et al., 2010).

## Combatting consequences of spaceflight, muscle disuse, disease, and aging using YoYo™

Astronauts may experience lower limb muscle loss amounting to 10% in the first month of spaceflight (LeBlanc et al., 1995; Tesch et al., 2005). In parallel, loss of bone mineral density amounts to ~1% monthly during lack of weight bearing (Shackelford et al., 2004; Sibonga et al., 2007). Similar to the impact of the *0-g* environment, injuries, diseases or other conditions leading to severely reduced levels of physical activity and skeletal muscle unloading, exert immediate, obvious negative effects on muscle size, function and integrity. It is worth noting that marked skeletal muscle protein breakdown and associated molecular changes are apparent after only 3 days of unilateral lower limb suspension (ULLS; an established spaceflight analog to study skeletal muscle). Muscle unloading (Tesch et al., 2016) resulting from withdrawal of weight-bearing activities alters expression of pathways and markers for contractile protein breakdown and synthesis (Tesch et al., 2008; Gustafsson et al., 2010; Reich et al., 2010). Such compromised protein metabolism appears to facilitate significant muscle loss within 5 days (Wall et al., 2014). In clinical practice, this would imply that exercise countermeasures aimed at combatting negative consequences of muscle disuse should be implemented as early as medically possible. Thus, the efficacy of YoYo™ exercise training should be explored in patients and populations at obvious risk of suffering from muscle deconditioning.

### Lower limb unloading and bed rest

Quadriceps muscle volume decreased by 9% in men and women subjected to 5 weeks of ULLS. In parallel, muscle strength decreased by ~30%. In contrast, when concurrently employing YoYo™ resistance exercise twice or thrice weekly, offering only 16 min contractile activity over the course of this intervention, muscle volume rather showed an impressive 8% increase (Tesch et al., 2004b). Similarly, the marked strength loss noted after ULLS only was offset. At the molecular level, there were reductions in total RNA as well as in actin and myosin mRNA expression following ULLS. These changes were counteracted by YoYo™ knee extensor exercise (Haddad et al., 2005). Similarly, ULLS decreased PGC-1alpha, and increased phosphofructokinase expression (Fernandez-Gonzalo et al., 2014a). Despite the limited amount of work produced, these effects were offset by concurrent YoYo™ exercise. This suggests that skeletal muscle metabolic perturbations, and most notable the reduced oxidative capacity, could be attenuated by bouts of high-force, low-volume YoYo™ resistance exercise.

Furthermore, 29 and 90 days' bed rest decreased quadriceps muscle size by 10 and 18%, respectively. This marked atrophy was offset when YoYo™ exercise (supine squat; 4 sets × 7 reps) was carried out every third day (Alkner and Tesch, 2004a,b). Long-term bed rest also compromised motor control functions. Thus, the EMG amplitude required to produce a certain force task, or sustain an isometric action, showed noticeable increases (Alkner et al., 2016). These investigators also reported reduced rate of force development following bed rest. Most importantly however, all these effects were abolished in individuals who concurrently performed YoYo™ exercise. It is also worth noting that heel raises and supine squats performed every third day using YoYo™ was equally effective as an established pharmaceutical (Pamidronate) intervention against bone loss, to blunt the decrease in bone mineral density experienced after 90 days' bed rest (Rittweger et al., 2005). Further, while m. vastus lateralis collagen/muscle protein content ratio increased in response to bed rest, it remained unchanged in individuals who performed concurrent YoYo™ exercise (Nielsen et al., 2016). This exercise regimen also counteracted some, but not all, muscle metabolic perturbations induced by the 90 days' bed rest (Irimia et al., 2017).

Altogether, prescribing YoYo™ exercise, employing eccentric overload, appears highly effective in combating muscle atrophy and dysfunction and metabolic perturbations. The method may also attenuate loss or alterations of other tissues impacted by long-term disuse or lack of weight bearing. Short-term interventions (<3 weeks) employing resistance exercise using weights (not feasible in *0-g*) as countermeasures also abolish strength and muscle loss (Schulze et al., 2002). There are no long-term bed rest studies available employing feasible resistance exercise methods for *in-flight* use that have explored in detail adaptations proving efficacy in counteracting neuromuscular dysfunction occurring during spaceflight.

### Aging

Resistance exercise training is now an established method of treating age-induced skeletal muscle atrophy, i.e., sarcopenia. YoYo™ exercise appears to serve this cause more effectively than that noted with traditional weight training. Thus, in ~70-year old men and women, 12 weeks YoYo™ knee extensor training increased isokinetic power by 28%. This effect was significantly greater than noted in age-matched individuals who trained with weights (Onambele et al., 2008). Likewise, YoYo™ but not weight-stack exercise, improved single-leg balance. The authors attributed this response to increased tendon stiffness and neuromuscular transfer/overspill to the plantar-flexors in those who trained with YoYo™. This exercise task also called for significantly higher overall EMG activity. Further, increases in muscle volume (5%) and isometric and isotonic force (~10%) were noted following 8 weeks YoYo™ Leg Press exercise combined with high-intensity aerobic training in 70-year-old healthy men (Bruseghini et al., 2015). While this study did not investigate conventional weight training, reports comparing eccentric and conventional resistance training in older adult show no differential effect on muscle size and strength across exercise modes.

As resistance exercise remains the most established, yet underutilized, method to treat age-induced skeletal muscle loss, we urge the use of YoYo™ exercise in future investigations aimed at exploring adaptations of resistance exercise in the physically fit, but also in the impaired and dysfunctional elderly. Amid caution must be exercised when subjecting frail or impaired populations to this method there is strong support these individuals benefit more from high- than low-intensity exercise (Peterson et al., 2010).

## Injury prevention and rehabilitation

The efficacy of YoYo™ exercise to prevent hamstring injury was examined in 30 soccer players of the Swedish premiere league (Askling et al., 2003). While half of the players, in addition to their prescribed pre-season training routines, performed hamstring exercise for 10 weeks using the YoYo™ Leg Curl, the other players did not. Over the course of the competitive season, hamstring strain injury occurrence was significantly lower in the training group (3/15) than in players who did not receive this treatment (10/15). In support, de Hoyo and collaborators noted that there was a likely reduction in the number of days of absence per injury, and a possible drop in total injury incidence in young soccer players subjected to concurrent YoYo™ Squat and Leg Curl training (de Hoyo et al., 2015). Thus, it appears the improved vertical and linear speed or power demonstrated with this program (see above) was accompanied by reduced hamstring injury incidence and severity. Although, it remains to be established whether these effects are more substantial than those realized with other forms of accentuated eccentric loading paradigms, these findings suggest that YoYo™ exercise not only benefits athletic performance but could also serve as an important adjunct to combat hamstring muscle strain, and perhaps other soft-tissue injuries.

The effects of open chain knee extensor exercise, using either YoYo™ or a weight-stack machine were compared in individuals with a history of knee ligament injury (Greenwood et al., 2007). The YoYo™ paradigm was equally, if not more effective as the more conservative approach to improve muscle strength and size, neural activation, standing balance, and vertical jump performance. Similarly, athletes with chronic patellar tendinopathy who completed a 6-week training program employing the closed chain YoYo™ Leg Press, showed an impressive (90%) increase in eccentric strength. More significantly, clinical measures showed reduced pain and augmented tendon function. The results of this study suggest that short-term training using inertial eccentric overload has significant and beneficial effects on muscle function and pain in individuals suffering from long-lasting patellar tendinopathy (Romero-Rodriguez et al., 2011).

Athletes such as volley- and basketball players are at high risk of developing patellar tendinopathy. Given the paradox that eccentric training elicits performance and therapeutic benefits, yet might provoke such injury, prescribing exercise to these athletes is a delicate balancing act. Hence, in a subpopulation of 81 male and female players who carried on their normal in-season training routines over 24 weeks, a single bout YoYo™ Squat was prescribed once weekly (Gual et al., 2016). Neither group suffered from increased symptoms of patellar tendinopathy. Yet, increases in vertical jump, strength and power were most evident in those athletes receiving the high-intensity bout. It appears, therefore, that adding a weekly YoYo™ Squat bout to the prescribed regular routine enhances lower limb muscle power, without causing patellar tendon complaints. Similarly, 41 recreational or competitive athletes suffering from patellar tendinopathy were prescribed YoYo™ Leg Press exercise while treated with intra-tissue percutaneous electrolysis. According to the authors, only a few sessions of this combined paradigm allowed patients to return to previous physical activity levels as they experienced markedly improved knee function (Abat et al., 2014). Further, calf raise exercise training performed using the YoYo™ Squat, increased Achilles tendon cross-sectional area as well as the pennation angle of the medial aspect of the gastrocnemius muscle (Sanz-Lopez et al., 2016). Albeit there were no clinical outcome measures reported, these results suggest that eccentric overload training with YoYo™ Squat promotes muscle and tendon adaptations that could benefit athletic performance and serve to prevent injury. This accords with a wealth of clinical studies demonstrating the efficacy of eccentric exercise training as an aid in treating tendon disorders (O'Neill et al., 2015).

## Physiological effects of concurrent iso-inertial aerobic and YoYo™ resistance exercise

While resistance exercise effectively combats muscle atrophy and dysfunction, it does not prevent cardiovascular deconditioning. Astronauts suffer from both skeletal muscle and cardiovascular deconditioning while in orbit. Yet, future space vehicles on missions to distant planets will only carry a single exercise apparatus with the objective of preserving both muscle and cardiovascular function. To meet this challenge, our laboratory recently introduced a YoYo™ configuration (RAD; Resistance and Aerobic exercise Device) accomplishing concentric rowing exercise as an additional, sole aerobic feature (Tesch et al., 2013). In a proof-of-principle study (Owerkowicz et al., 2016), seventeen men and women completed 5 weeks of training employing the YoYo™ RAD. Supine Squats (4 sets of 7 reps) were performed twice weekly, and aerobic rowing (4 × 4-min) at ~90% VO_2max_ three times weekly. VO_2max_ (8%), 3-RM strength (18%), and quadriceps femoris cross-sectional area (10%), as well as fatigue resistance of the knee extensor muscles increased significantly. At the cellular and molecular level, muscle fiber size, citrate synthase activity, total RNA concentration, IGF-I mRNA, and Type IIa MHC mRNA showed marked increases. These functional outcomes and biomarker changes imply that low-volume concurrent YoYo™ aerobic and resistance exercise results in cardiovascular and musculoskeletal benefits comparable to those noted with more established exercise modalities. Subsequently, this research team investigated 19 men and women who were subjected to 10 days simulated spaceflight (ULLS). Ten of these individuals performed additional exercise using this particular YoYo™ RAD paradigm. While ULLS had profound adverse effects on skeletal muscle molecular markers and *in-vivo* function, the exercise intervention mitigated these changes, and was in fact capable of ameliorating both muscle strength and VO_2max_ (Cotter et al., 2015).

These findings are in stark contrast to research carried out almost 40 years ago, raising questions about the compatibility of aerobic and resistance exercise (Hickson, 1980; Leveritt et al., 1999). Albeit the seminal work by Hickson deserves recognition, unfortunately the conclusions drawn from this single study have become dogma. Indeed, employing more accurate protocols and currently available imaging and molecular methods, and combining smart and thoughtfully, concurrent aerobic and resistance exercise training could in fact amplify certain established exercise responses in non-athletic populations (Lundberg et al., 2013; Murach and Bagley, 2016). In further support, the very robust strength increase in 70-year old men, noted in response to YoYo™ Leg Press training, did not attenuate favorable changes in aerobic fitness, body composition, or blood lipid (LDL cholesterol) profile, elicited by a preceding aerobic exercise intervention (Bruseghini et al., 2015). Thus, YoYo™ training appears not to interfere with the beneficial effects of aerobic training in healthy senior men. This accords our demonstrations of important skeletal muscle adaptations following iso-inertial endurance exercise combined with YoYo™ resistance exercise training in healthy, young men (Lundberg et al., 2013, 2014). Hence, concurrent aerobic and resistance exercise may boost skeletal muscle hypertrophy (Mikkola et al., 2012; Lundberg et al., 2013), and may, in fact, produce positive effects on lean body mass and cardiovascular health markers similar or more profound than those evoked by aerobic or resistance exercise alone (Sillanpaa et al., 2010; Johannsen et al., 2016). In view of this, we are inclined to state that sedentary or recreationally active individuals can enjoy the benefits of YoYo™ exercise programs combining strength, power and endurance exercise of postural weight bearing muscles without compromising the effects of either mode when executed alone. It remains to be investigated if concurrent aerobic and resistance exercise protocols, employing other exercise modalities, elicit responses on par with the above findings.

## Neurological rehabilitation

Evidently, eccentric actions *per se* play a predominant role in producing increases in muscle strength, power, and size (Dudley et al., 1991b; Hather et al., 1991; Hortobagyi et al., 1996). This may be because eccentric actions rely on unique neural strategies to facilitate and evoke motor action potentials and bring motor units into play (Enoka, 1996; Hedayatpour and Falla, 2015). Further, with use of brain functional MRI, it has been shown that eccentric actions engage more functional regions of the brain than their concentric counterparts (Fang et al., 2004). Thus, eccentric actions are important in both central and peripheral nervous system adaptations in response to resistance exercise training. The first study assessing the feasibility of YoYo™ Leg Press exercise as a rehabilitation intervention for neurologically-impaired stroke patients, reported robust gains in muscle power and force after 8 weeks of training, accompanied by significant improvements in balance and gait (Fernandez-Gonzalo et al., 2014c). Spurred by these findings, Fernandez-Gonzalo and associates designed and conducted a more comprehensive randomized clinical trial assessing cognitive performance along with changes in quadriceps muscle volume, force, power, and daily life functions in chronic stroke patients subjected to 12 weeks YoYo™ Leg Press training (Fernandez-Gonzalo et al., 2016a). The results of this trial confirm and highlight the efficacy of this particular exercise paradigm to aid stroke patients regaining muscle mass, strength, and critical daily physical functions. However, while 12-week resistance exercise programs using concentric and eccentric actions by means of weights may produce similar results in stroke patients (Ryan et al., 2011), they would require at least six times the number of repetitions to be performed. Most remarkable, the exercise intervention (28 reps twice weekly), which called for less than 4 min of contractile activity per week (Fernandez-Gonzalo et al., 2016a), also improved vital cognitive functions such as executive performance, attention, and speed of information processing, suggesting YoYo™ exercise evoked paramount central nervous system adaptations. Such a response would be commensurate with the demonstration of muscle action-specific involvement of certain areas of cortex (Fang et al., 2001; Hedayatpour and Falla, 2015). Those findings are encouraging and should be followed by studies using, e.g., brain fMRI to discern the specific mechanisms and areas of cortex targeted with this unique exercise stimulus. Clinical trials in individuals suffering from other neurological pathologies are warranted to justify YoYo™ exercise as a viable and safe rehabilitation method.

## Guidelines for YoYo™ exercise training

The standard YoYo™ exercise protocol (4 sets of seven repetitions; 90–180 s rest between sets) should be performed no more than twice weekly with at least 48 h recovery between sessions. More frail individuals or populations suffering from disorders should be allowed the full 180 s rest between sets. In patients undergoing rehabilitation from injury or trauma and concurrently subjected to other exercise regimen(s), YoYo™ exercise performed once weekly is advised. In healthy individuals subjected to endurance and resistance exercise training of the same muscle group on the same day, any all-out aerobic exercise session should be performed at least 6 h prior to YoYo™ exercise. In more frail populations, caution and careful tracking and monitoring of performance (e.g., power), in each session and over time, is necessary to ensure full recovery and uncompromised performance. If training for power is preferred, lower inertia should be chosen. Should training rather target force development, high inertia should be chosen. Training progress should occur by encouraging the trainee to delay the breaking eccentric action, which results in increased eccentric overload. High training volume or frequency is not a necessity. As there is limited knowledge about responses to YoYo™ exercise using upper-body muscles, the above guidelines apply to exercise involving weight-bearing and lower-limb muscles.

## Side effects and safety issues

Feasibility and safety issues employing various YoYo™ configurations, e.g., open- or closed chain knee extensions, leg curl, vertical or supine squat, calf raise, back extension, arm curl or row, have been reported in studies of professional athletes, recreationally active, sedentary or disabled individuals of either sex and ranging in age from young adults to +90-year-old men. Collectively, in these individuals, being ambulatory or subjected to muscle unloading, long-duration (up to 110 day) confinement or bed rest, or returning from space, no serious muscle-tendon injury or severe muscle soreness (delayed onset of muscle soreness; DOMS) or complaint has been reported during or following YoYo™ use.

It is generally held that novel eccentric exercise provokes muscle damage and subsequent inflammatory symptoms, resulting in DOMS (Newham et al., 1983; Hortobagyi et al., 1998; Krentz and Farthing, 2010; Fernandez-Gonzalo et al., 2012). Thus, established skeletal muscle “damage markers” were assessed in 32 men and women at onset of, and following the very last training session of a 6-week program comprising 2–3 weekly bouts using the YoYo™ supine squat (Fernandez-Gonzalo et al., 2014b). While creatine kinase and lactate dehydrogenase levels were elevated after novel YoYo™ exercise, this response was not evident after the very last bout. Still, muscle power was ~50% greater than noted initially (Fernandez-Gonzalo et al., 2014b). Taken together, muscle damage is not likely to interfere with desired exercise-induced muscle adaptations evoked by YoYo™ exercise training due to the protective effect from repeated training bouts. Nonetheless, supervised, familiarization (≥3 sessions) using progressively increased effort, and hence load, is recommended to ensure good form, comfort, and consistent performance to reduce DOMS or injury risk. Individuals employing this strategy typically showed steady progression in force and power across sessions in the course of short- (Lundberg et al., 2013) or long-term (Alkner et al., 2003) training programs, with maintained or increased self-reported comfort. Notwithstanding, when introduced to novice or clinically frail individuals, caution should be exercised to allow for complete recovery between sessions and ensure effort is progressive. Thus, excess eccentric exercise may be counterproductive or even injurious to certain populations and may not be prescribed unconditionally to patients suffering from muscle- or neurological disorders. However, people recovering from stroke coped well with the maximal nature of YoYo™ exercise. In fact, patients showed 100% adherence to the protocol referred to above and improved peak power at a weekly rate of ~5% without experiencing DOMS or increased muscle spasticity (Fernandez-Gonzalo et al., 2014c, 2016a). The safety, feasibility and efficacy of YoYo™ to serve in neurological rehabilitation are further evident in patients suffering from multiple sclerosis and Alzheimer's disease. Hence, after complying with a 12-week intervention comprising YoYo™ Squat exercise (Sarmiento et al., 2014; Oliveira et al., 2015) these individuals demonstrated markedly improved gait performance and muscle strength with no severe medical complications.

## Summary and future perspectives

Chronic exercise training using YoYo™ produces early, robust neuromuscular adaptations, which appear to be more pronounced than noted with traditional weight training. Improvements in strength and power stem from preeminent skeletal muscle hypertrophy and increased neuronal activity, inferring the unique iso-inertial loading strategy typical of YoYo™ exercise offers a highly potent stimulus to optimize the benefits of resistance exercise. The method is in the advent of clinical use. Although there is strong support that this exercise paradigm could improve function in patients suffering from, e.g., neurological pathologies, age-induced sarcopenia or musculo-tendinous problems, there is call for fine-tuning and exploring individualized protocols to be prescribed for men and women with clinical symptoms. Validating and revising currently employed protocols and prescriptions with regard to, e.g., exercise frequency, volume and/or inertia for individual and disease-specific use, would assure larger populations could safely entertain the benefits of YoYo™ exercise training. Due to the time constrains of current institutional health care and rehabilitation systems, there is a need to define the minimum dose required to induce the desired beneficial effects prompted by this exercise paradigm. Thus, studies of different clinical populations, comparing the efficacy of YoYo™ and other exercise methods, are warranted to define protocols optimizing exercise benefits and ensure patients are not “under-loaded” or at risk of being “over-loaded.”

**Figure 2:**
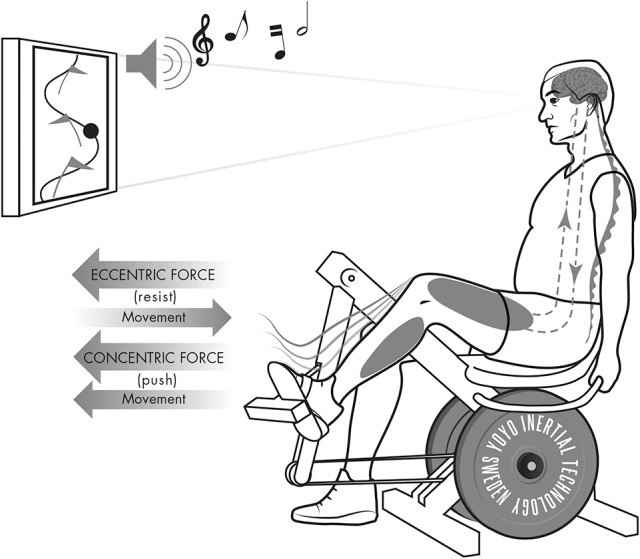
**Cartoon depicting YoYo™ Leg Press employed in a virtual reality environment**. Full arrows highlight the direction of the muscle force during concentric and eccentric actions vs. the direction of the movement; dashed arrows represent the cross-talk between the central nervous system and the skeletal muscle.

The demonstration of important neuropsychological benefits induced by YoYo™ exercise in stroke patients deserves attention. We urge investigators to conduct research addressing the underlying mechanisms incurring such adaptations. Likewise, as therapeutic aids to improve cognitive functions are routinely used in rehabilitation following brain trauma, the potential benefits or drawbacks of employing YoYo™ exercise in a virtual reality environment should be explored. Based on our studies encompassing stroke victims, we believe protocols offering simultaneous neural-, muscle- and cognitive activation, could present a contingent challenge and valuable asset in neurological rehabilitation (Figure 2).

YoYo™ exercise protocols should be tested in other not explored, clinical conditions characterized by muscle loss and impaired neuromuscular function, and/or dysfunctional endocrine or metabolic control. Examples of such conditions, which interfere with daily living physical abilities, include, e.g., cancer, cachexia, burns, kidney failure, heart failure, muscle dystrophies, chronic alcoholism, and various autoimmune diseases. Further research in various patient categories, exploring physiological adaptations to YoYo™ exercise in comparison with other forms of resistance training, could aid in generating new effective exercise therapies to combat dysfunction while improving quality of life. Until then, extrapolating results from currently available studies of healthy men and women subjected to YoYo™ exercise, to non-explored clinical conditions should be done with caution. Notwithstanding, with the current information at hand it appears YoYo™ exercise serves as a useful tool for improving neuromuscular function in both healthy and clinical populations.

## Author contributions

Conception and design of the work: PT, RF, and TL. Drafting the work and revising it critically for important intellectual content: PT, RF, and TL. Final approval of the version to be published: PT, RF, and TL. Agreement to be accountable for all aspects of the work in ensuring that questions related to the accuracy or integrity of any part of the work are appropriately investigated and resolved: PT, RF, and TL.

## Funding

Research employing YoYo™ conducted by PT and referred to herein, have been funded by grants from the National Astronautics and Space Administration (NASA), the National Space Biomedical Research Institute (NSBRI, the European Space Agency (ESA), the Swedish National Space Board (SNSB) and the Swedish Research Council for Sport Science (CIF).

### Conflict of interest statement

PT and Hans Berg founded YoYo Technology AB (Inc.) of Stockholm, Sweden in year 1993. PT is a shareholder of YoYo Technology AB and YoYo™ Sports and Medical sl, which control the immaterial rights to YoYo™. The other authors declare that the research was conducted in the absence of any commercial or financial relationships that could be construed as a potential conflict of interest.
